# Summary of *BARD1* Mutations and Precise Estimation of Breast and Ovarian Cancer Risks Associated with the Mutations

**DOI:** 10.3390/genes11070798

**Published:** 2020-07-15

**Authors:** Malwina Suszynska, Piotr Kozlowski

**Affiliations:** Department of Molecular Genetics, Institute of Bioorganic Chemistry, Polish Academy of Sciences, Z. Noskowskiego St. 12/14, 61-704 Poznan, Poland; msuszynska@ibch.poznan.pl

**Keywords:** *BARD1*, breast cancer, cancer risk, meta-analysis, mutation, ovarian cancer, pathogenic variant

## Abstract

Over the last two decades, numerous *BARD1* mutations/pathogenic variants (PVs) have been found in patients with breast cancer (BC) and ovarian cancer (OC). However, their role in BC and OC susceptibility remains controversial, and strong evidence-based guidelines for carriers are not yet available. Herein, we present a comprehensive catalog of *BARD1* PVs identified in large cumulative cohorts of ~48,700 BC and ~20,800 OC cases (retrieved from 123 studies examining the whole coding sequence of *BARD1*). Using these resources, we compared the frequency of *BARD1* PVs in the cases and ~134,100 controls from the gnomAD database and estimated the effect of the *BARD1* PVs on BC and OC risks. The analysis revealed that *BARD1* is a BC moderate-risk gene (odds ratio (OR) = 2.90, 95% CIs:2.25–3.75, *p* < 0.0001) but not an OC risk gene (OR = 1.36, 95% CIs:0.87–2.11, *p* = 0.1733). In addition, the *BARD1* mutational spectrum outlined in this study allowed us to determine recurrent PVs and evaluate the variant-specific risk for the most frequent PVs. In conclusion, these precise estimates improve the understanding of the role of *BARD1* PVs in BC and OC predisposition and support the need for *BARD1* diagnostic testing in BC patients.

## 1. Introduction

Inherited genetic factors are responsible for a substantial portion of breast cancer (BC) and ovarian cancer (OC) cases. Multigene panel testing has revealed that germline loss-of-function mutations/pathogenic variants (PVs) in various cancer-associated genes are identified in more than 10% and 20% of patients with BC and OC, respectively [1,2,3]. Attempts have been made to define the genetic/familial risk of BC/OC associated with these genes, and subsequently, management recommendations for carriers of PVs in some of the high-penetrance genes have been established [4,5]. Despite this, several genes in which PVs confer low- or moderate-penetrance effects still require more evidence and more convincing assessments of BC/OC risk to utilize them in recommendations for carriers. A group of genes with insufficient and/or conflicting data includes the *BRCA1*-associated *RING domain 1* (*BARD1*) gene.

The *BARD1* gene encodes a protein of 777 amino acids with several functional domains, including an N-terminal RING-finger domain, three ankyrin repeats (ANK), and two C-terminal BRCT domains. The protein shows structural homology with BRCA1 within the BRCT and RING-finger domains, and through the latter domains, the two proteins form a stable heterodimer. The BARD1-BRCA1 heterodimer has E3 ubiquitin ligase activity and acts in multiple cellular processes essential for maintaining genomic stability, including DNA double-strand break repair through homologous recombination [6,7,8]. In addition to the well-established role of the BARD1-BRCA1 heterodimer, the BRCA1-independent function of BARD1 as a tumor suppressor has also been postulated, for example, in mediating p53-dependent apoptosis [9]. On the other hand, several BARD1 isoforms resulting from alternative splicing that lack RING-finger and/or ANK domains were upregulated in different cancers and are suggested to have an oncogenic effect by antagonizing the function of full-length BARD1 [10,11,12,13].

Because of the abovementioned BARD1 functions, many mutation screening studies have been carried out to explore the role of *BARD1* in BC and OC susceptibility. As a result, hundreds of PVs were detected, mostly through the use of high-throughput next-generation sequencing (NGS; panel sequencing) in recent years. However, the PVs are scattered among many articles, which prevents the drawing of more comprehensive conclusions about their distribution over the gene, identification of recurrent PVs, and estimation of a more precise effect on cancer risk. In addition, despite multiple examples of *BARD1* PVs in BC/OC patients, attempts to link PVs with BC/OC risk are often inconclusive and are usually not supported by statistically convincing pieces of evidence. However, these types of studies are justified due to reduced mutation penetrance and various interactions between the gene and other genetic, personal, and environmental factors. The lack of proper functional characterization of PVs and the genetic heterogeneity in different populations may be additional factors of insufficient and/or conflicting data. In addition, due to the low frequency of *BARD1* PVs (<1%), the sample sizes of the studies are still too modest to achieve sufficient statistical power, although the sizes are constantly increasing. Additionally, as the cost of whole gene testing is still relatively high and association case-control studies relying on the use of geographically matched controls are still very rare [14,15,16,17]. Therefore, controls from publicly available databases are frequently used to assess the risk, allowing to increase the study size and to improve statistical power [3,18,19,20]. Cosegregation studies can provide additional evidence for or against the association, although they may be challenging for low or moderate penetrance PVs, especially because large families with multiple BC/OC cases are becoming less available.

To overcome the limitations mentioned above, in this study, we took advantage of the effort made by scientists in over 20 years of *BARD1* gene screening and cataloged all the published *BARD1* PVs identified in 123 studies in a total of ~48,700 BC and ~20,800 OC patients. The compilation can serve as a resource of information on *BARD1* germline PVs and their frequencies. Using the collected data, we determined with high confidence the overall BC and OC risk associated with PVs in *BARD1*. As some PVs might confer different risks than overall gene-specific risk, we also evaluated the variant-specific risk for the most frequent PVs.

## 2. Methods

### 2.1. Literature Search and Study Selection

A systematic search of PubMed was performed for all studies published until April, 2020, that analyzed the *BARD1* gene in BC and OC patients. The study adhered to the PRISMA guidelines. Combinations of the following search terms were used: “BARD1, breast cancer, ovarian cancer, multi-gene/multigene panel, mutation, variant, germline, susceptibility, predisposition”. Two authors (MS and PK) independently examined the titles and abstracts of the records and selected eligible studies. Additional studies were identified through handsearching, e.g., by examining reference lists of included studies. Studies screening the whole coding sequence of *BARD1* in BC and OC patients were eligible for analysis and were included independent of BC/OC tumor subtype and BC/OC family history of patients. All studies with incomplete data, e.g., lacking the number of BC/OC patients, lacking a list of identified *BARD1* PVs, or analyzing only selected *BARD1* PVs or exons, were excluded. Studies that used the overlap cohort of patients for multiple publications were also excluded, except for the most complete and/or extensive articles.

### 2.2. Data Extraction and Analysis

Information regarding germline loss-of-function PVs (i.e., frameshift, nonsense, ±1/±2 position splicing variants) and missense variants described as pathogenic/likely pathogenic in the ClinVar database [21] (https://www.ncbi.nlm.nih.gov/clinvar/), as well as the number of patients tested, was extracted from all included studies. In the case of BC/OC familial studies, BC and OC patients were separately included in either the BC or OC group, respectively; patients with both cancers (BC and OC) were included twice in both groups. Information on geographic origin, methodology used, BC/OC family history status, and prior *BRCA1*/*2* testing was noted. The overall mutation frequency in *BARD1* and the frequency of each PV were calculated by pooling data (the number of PVs/the number of analyzed patients) of all selected studies (separately for the BC and OC groups). The overall and variant-specific frequencies in controls were calculated using data extracted from the noncancer cohort of the Genome Aggregation Database (gnomAD) [22,23] (https://gnomad.broadinstitute.org/), as of April, 2020. As in the case group, only loss-of-function PVs and pathogenic/likely pathogenic missense variants were taken into account in the controls. Using a single comparison of mutation frequency in cases and controls, the overall (gene-specific) and variant-specific BC and OC risks were estimated. To gather the largest sample size for association analyses, data from all studies and all controls, representing multiple geographic areas, were used. However, as mutation frequencies may vary in different populations, the risk was also estimated separately for major populations tested, i.e., Caucasians and Asians, using adequate population controls from the gnomAD database. The predominant ethnicity in selected studies was determined based on the country/origin of publication and/or ethnicity information indicated in the study. Studies from the USA, with the Caucasian population below 80% or not reporting ethnicity at all, were excluded from the association analysis carried out for the Caucasian population. The association analyses were repeated without studies containing the results of multigene testing in samples submitted to diagnostic companies and without studies using older technologies (i.e., high-resolution melting, conformation-sensitive gel electrophoresis, denaturing gradient gel electrophoresis, denaturing high-performance liquid chromatography, and Sanger sequencing) to estimate the potential bias. Additional association analysis was carried out for studies that analyzed patients with a family history of BC/OC (at least 90% of patients positive for a BC/OC family history). Associations between PVs in *BARD1* and BC/OC risk were assessed using odds ratios (ORs) and 95% confidence intervals (95% CIs) based on the chi-square test. All statistical tests were two-sided, and a *p*-value of less than 0.05 was considered statistically significant. Analyses were conducted using MedCalc Statistical Software version 14.8.1 (MedCalc Software bvba, Ostend, Belgium; http://www.medcalc.org).

## 3. Results

### 3.1. Eligible Studies

To identify published studies reporting germline PVs in *BARD1* in BC/OC patients, we searched studies indexed in PubMed up to April, 2020. As presented in the flow chart of study inclusion (Supplementary Figure S1), the search initially identified 9151 records, from which 8886 records were excluded based on title and abstract review. A total of 265 studies were further assessed by the full-text review, of which 145 studies not meeting the criteria (described in the Methods section and Figure S1) were excluded. Finally, for the analysis, we included 123 studies: 120 studies from the PubMed search and 3 hand-searched studies [15,16,18,19,24,25,26,27,28,29,30,31,32,33,34,35,36,37,38,39,40,41,42,43,44,45,46,47,48,49,50,51,52,53,54,55,56,57,58,59,60,61,62,63,64,65,66,67,68,69,70,71,72,73,74,75,76,77,78,79,80,81,82,83,84,85,86,87,88,89,90,91,92,93,94,95,96,97,98,99,100,101,102,103,104,105,106,107,108,109,110,111,112,113,114,115,116,117,118,119,120,121,122,123,124,125,126,127,128,129,130,131,132,133,134,135,136,137,138,139,140,141,142]. A total of 105 and 56 of the studies contained data regarding BC and OC patients, respectively. The studies were conducted in different geographic locations, including BC/OC patients of various ethnicities, with a predominance of European and, then, Asian populations. The characteristics of the included studies are listed in Supplementary Table S1.

### 3.2. BARD1 Mutational Spectrum

**Figure 1:**
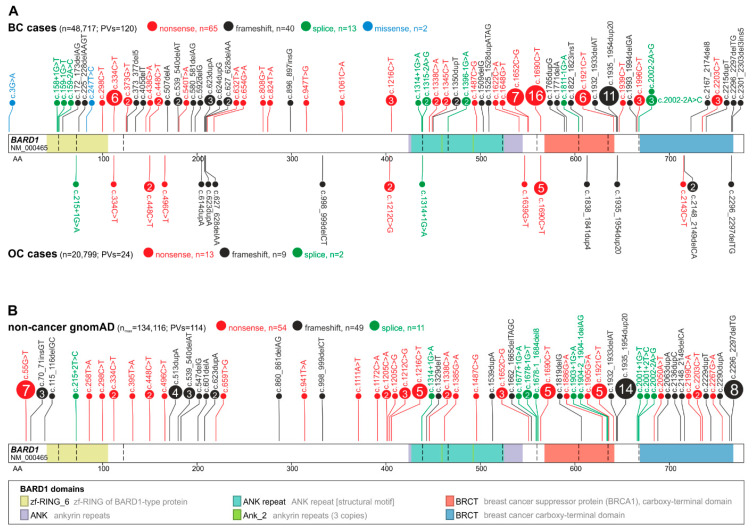
Maps of deleterious PVs in *BARD1*. PVs are shown alongside the *BARD1* coding sequence with the indicated exon structure and the protein functional domains. The size of a PV symbol (circle) is proportional to the number of PVs, and color indicates the type of PV. (**A**) PVs detected in BC (above) and OC (below) cases. (**B**) PVs reported in noncancer gnomAD controls. The total number of detected PVs and the total number of cases and controls tested for the variants are indicated in parentheses; note that the total number of tested subjects differs substantially between groups.

In total, 144 *BARD1* PVs, constituting 69 distinct PVs, were identified in either BC or OC cases, constituting 120 PVs (60 distinct PVs) in 48,717 BC cases (0.25%) and 24 PVs (17 distinct PVs) in 20,799 OC cases (0.12%). Out of 69 distinct PVs in the BC and OC cohorts, 21 were present in population controls. The distributions of mutation types were similar in BC and OC, with nonsense, frameshift, splicing, and pathogenic missense variants accounting for 54%, 33%, 11%, and ~2% in BC and 54%, 38%, 8%, and 0% in OC, respectively. For comparison, in population controls, nonsense, frameshift, and splicing variants accounted for 47%, 43%, and 10%, respectively. The distribution of all the PVs in *BARD1* detected in the BC and OC cohorts, as well as in population controls, is presented in Figure 1.

Although *BARD1* PVs in BC and OC patients are distributed over the entire coding sequence, there are two regions of increased density of PVs, i.e., from exons 2 to ~230AA in exon 4, overlapping the RING-finger domain and from exon 5 to exon 10, overlapping the ANK repeat and BRCT I domains.

In the BC cohort, 10 of the PVs were identified in three or more cases (defined here as recurrent), with the highest occurrence of c.1690C>T (Q564*), c.1935_1954dup20 (E652Vfs*69), c.1652C>G (S551*), c.334C>T (R112*), and c.1921C>T (R641*) reported in 16, 11, 7, 6, and 6 cases, respectively. R112* and R641* recurrent PVs were identified in both Caucasian and Asian populations, while the remaining PVs, including the most frequent, Q564* and E652Vfs*69, were identified only in the Caucasian population. None of the recurrent PVs were specific to the Asian population. Interestingly, 10 out of 16 Q564* PVs were reported by studies analyzing mainly patients with a family history of BC/OC. Only one recurrent PV, i.e., Q564*, was reported in the OC cohort (five patients). In population controls, an additional recurrent PV was observed (not identified in BC/OC cohorts), i.e., c.55G>T (E19*). However, the PV was characteristic only for the Latino population, which represents a small proportion of the entire population of BC/OC cohorts.

### 3.3. Association of BARD1 Pathogenic Variants with Breast Cancer

**Figure 2:**
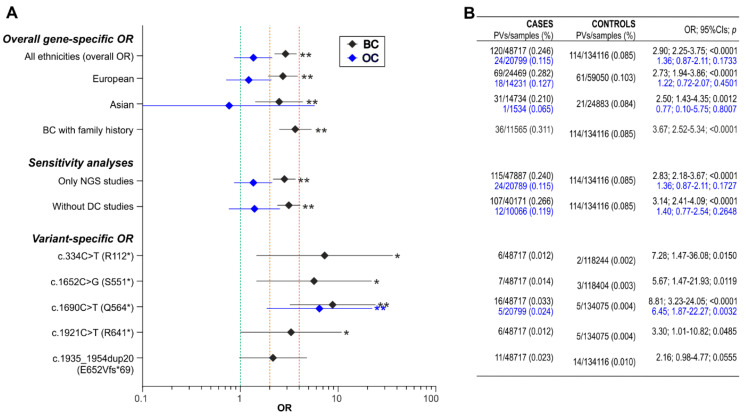
Summary of the BC and OC risk (OR) associated with *BARD1* PVs. (**A**) The graph showing gene-specific and variant-specific ORs. The gene-specific OR is provided for all ethnicities combined and separately for European and Asian populations. “Only NGS studies” and “Without DC studies” demonstrate the results of repeated association analyses without studies applying older technologies and without studies publishing results of multigene testing in samples submitted to diagnostic companies, respectively. Diamonds and horizontal lines indicate the OR values and 95% CIs, respectively. Black and blue symbols represent BC and OC, respectively. Green, orange, and red vertical lines highlight an OR of 1 (no risk), an OR of 2 (the threshold for moderate risk), and an OR of 4 (the threshold for high risk), respectively. * or ** next to the OR symbol indicates a *p*-value < 0.05 or < 0.01, respectively. (**B**) The table showing the exact numbers and percentages (in the bracket) of detected PVs in either BC (black fonts) or OC (blue fonts) cases and in controls, as well as ORs with 95% CIs and *p*-values (the values correspond to the particular OR symbols shown in the graph on the left).

The prevalence of *BARD1* PVs was higher in BC patients (0.25%) than in population controls (0.09%), with a cumulative OR = 2.90 (95% CIs:2.25–3.75; *p* < 0.0001), classifying *BARD1* as a moderate BC risk gene. The risk was slightly higher for familial BC patients (OR = 3.67; 95% CI:2.52–5.34; *p* < 0.0001), evaluated based on data from studies that mainly analyzed cases with a BC/OC family history. To check whether the mixed population affected the results, similar association analyses were performed separately for Caucasian and Asian populations. As shown in Figure 2, BC risk estimates for Caucasians and Asians do not differ substantially, and both are only slightly lower than the risk estimates for mixed populations (OR = 2.73; 95% CIs:1.94–3.86; *p* < 0.0001 for Caucasians and OR = 2.50; 95% CIs:1.43–4.35; *p* = 0.0012 for Asians). We repeated the calculations excluding the three large studies from the analysis, which reported results of multigene testing of samples tested in diagnostic companies [125,139,141], as some small fractions of these samples may represent unrecognized duplicates of patients analyzed in other studies. Excluding these studies did not change the risk estimates substantially (OR = 3.14; 95% CIs:2.41–4.09; *p* < 0.0001). Moreover, to estimate the potential bias associated with the use of older technologies, which may be less sensitive when detecting some variants, we repeated the analysis including only studies reporting variants detected using NGS; again, the risk estimates showed no substantial difference (OR = 2.83; 95% CIs:2.18–3.67; *p* < 0.0001).

For five recurrent PVs, identified in at least five cases that have also been reported in controls, we calculated variant-specific ORs. Surprisingly, for 3 of these PVs, i.e., R112*, S551*, and Q564*, the variant-specific BC risk turned out to be substantially higher (OR > 4) than the overall gene-specific risk. The variant-specific risks of the other two recurrent PVs were in the range of the gene-specific risk, although it must be noted that due to the low number of particular PVs, the statistical power of the variant-specific risk estimations is generally low, and for one of the variants did not reach the nominal significance threshold (*p* = 0.05).

### 3.4. Association of BARD1 Pathogenic Variants with Ovarian Cancer

The prevalence of *BARD1* PVs was only slightly higher in OC patients (0.12%) than in population controls (0.09%), with a slight non-significantly increased cumulative OR = 1.36 (95% CIs:0.87–2.11; *p* = 0.1733). Therefore, the association was insufficient to classify *BARD1* as a low-risk gene for OC. The lack of association of *BARD1* PVs with OC was also supported by the analysis performed for extracted Caucasian and Asian populations, for which even lower ORs were obtained (OR = 1.22; 95% CIs:0.72–2.07; *p* = 0.4501 for Caucasians and OR = 0.77; 95% CIs:0.10–5.75; *p* = 0.8007 for Asians). The exclusion of the two largest studies reporting results from diagnostic companies and accounting for ~50% of cases [18,34] and limiting the analysis only to the studies using NGS did not affect the risk estimates (OR = 1.40; 95% CIs:0.77–2.54; *p* = 0.2648 and OR = 1.36; 95% CIs:0.87-2.11; *p* = 0.1727, respectively). Surprisingly, the variant-specific risk estimated for the most frequent OC PV, Q564*, suggests that the PV may be an OC high-risk allele (OR = 6.45; 95% CIs:1.87–22.27; *p* = 0.0032). This result, however, must be considered carefully, as it may result from the unequal proportion of Caucasian and Asiatic patients in OC and control populations. Indeed, as this PV is characteristic of the European population [14], the recalculation of the risk estimate presented a lower, yet still quite high, OR (OR = 3.32; 95% CIs:0.89–12.36; *p* = 0.0738).

### 3.5. Large Mutations in the BARD1 Gene

As the identification of large mutations (CNVs; deletions/duplications of single or several exons) in NGS data is a challenging task, CNV mutations were not reported in most of the selected studies. Therefore, we did not take them into account in the formal risk estimates in our study. Only five CNVs were identified in the *BARD1* gene in the selected studies. Deletion of exon 1, deletion of exon 2, and deletion of the entire gene (exons 1–11) were identified in BC patients [26,81,125], while deletion of exons 8–11 and deletion of the entire gene were identified in OC patients [34]. Of note, no large deletions were reported in the *BARD1* gene in gnomAD population controls (~10,800 cases tested), and only 1 deletion (exon 4–11) was identified among noncancer FLOSSIES controls (9884 cases tested). Comparing these numbers and considering the lack of CNV analysis in most included studies suggests that CNVs in *BARD1* are more frequent in BC/OC patients than in the general population and, therefore, most likely also contribute to BC/OC risk. However, as CNVs documented in *BARD1* account for a small fraction of all PVs in the gene, this observation is still insufficient to determine the real contribution of the *BARD1* CNV mutations to the risk of BC/OC.

## 4. Discussion

We performed a pooled analysis of the *BARD1* screening studies to catalog PVs identified in BC/OC patients over a 20-years period and to estimate a reliable *BARD1*-associated BC and OC risk. The association of *BARD1* with BC/OC has not been convincingly and unequivocally determined, and existing data, including our own [14,143], are still insufficient to ensure the solid interpretation of identified *BARD1* variants and make recommendations for carriers [4,5,144]. Defining the correct risk ratio for infrequently mutated genes, such as *BARD1*, whose PVs confer a low or moderate effect on phenotype, might be challenging; therefore, only large-scale studies can provide strong evidence.

In this study, we described a wide mutational spectrum of *BARD1*, encompassing 69 distinct PVs identified in 144 BC and OC patients. Generally, PVs were distributed over the whole *BARD1* coding sequence, although two regions of increased mutation density were also observed, the first overlapping the RING-finger domain and the second overlapping the ANK repeat and BRCT I domains. When comparing the PVs distribution between BC/OC patients and population controls, no evident hotspot characteristic of BC/OC could be defined. However, several recurrent PVs (*n* ≥ 5), which have been noted in BC patients, had a lower prevalence rate in population controls. Considering the generally dispersed distribution of PVs in *BARD1*, sequencing of the entire protein-coding region of the gene is recommended for diagnostic purposes; however, the genotyping of the most frequent PV, i.e., Q564*, may also have practical utility in central European populations [14,15].

The collection of ~48,700 BC and ~20,800 OC cases in the pooled analysis allowed us to achieve higher statistical power and more precisely estimate the overall BC and OC risk attributed to *BARD1* PVs than in previous individual studies. The prevalence of deleterious PVs in *BARD1* in the BC group (0.25%) was significantly higher than in population controls (0.09%); however, the frequency of PVs in the OC group (0.12%) was similar to that observed in controls. The pooled analysis of all studies revealed that PVs in *BARD1* were associated with a moderate BC risk (OR = 2.9), and only slightly lower ORs were obtained for European and Asian cases than population-matched controls. The results are in line with the risk estimates reported in other individual studies. Couch et al. reported an OR of 2.2 (*p* = 0.002; *n* = 28,536) for BC patients of European ancestry [3], Slavin et al. reported an OR of 3.2 (*p* = 0.012; *n* = 2127) for BC patients with a family history of BC [20], while our previous large-scale case-control study, based on genotyping of one recurrent PV, indicated an OR of 2.3 (*p* = 0.04; *n* = 13,935) [14]. The other studies did not confirm the association of *BARD1* PVs with BC or reported that increases in mutation frequencies were not significant [17,122,145], most likely due to an insufficient number of analyzed cases. Interestingly, Weber-Lassalle et al. reported a much stronger association (OR = 5.4; *p* < 0.00001; *n* = 4469) in BC patients with a family history of BC and an even higher risk (OR = 12.0; *p* < 0.00001; *n* = 782) for BC patients diagnosed under 40 years of age, suggesting that *BARD1* might be a risk gene for early-onset familial BC [15]. In our recent study, in which we performed a large-scale association analysis of central European founder PV, i.e., Q564*, we did not observe a higher risk for BC patients diagnosed under 40 years of age, however, the risk was elevated for triple-negative BC (OR = 3.6; *p* = 0.02; *n* = 1120) and bilateral BC (OR = 5.1; *p* = 0.02; *n* = 498), which are also indicators of hereditary BC [14]. Similar observations were reported in other studies [145,146,147], indicating that *BARD1* is a risk gene for familial BC. Consistently with the above, we observed a slightly increased risk for BC cases with a BC/OC family history.

In this study, we identified several recurrent PVs (potentially founder PVs in particular populations) that have been difficult to recognize in individual studies, and subsequently, we aimed to determine the variant-specific BC risk for these PVs. Surprisingly, three out of the five most frequent recurrent PVs were associated with substantially higher BC risk (OR > 5) than the overall risk estimated for the entire gene and two other recurrent PVs, which resembles an elevated risk associated with individual variants in other BC risk genes, e.g., the c.7271T>G (V2424G) variant in *ATM*, c.1036C>T (R346C) in *CHEK2*, and c.3113G>A (W1038*) in *PALB2* [148]. These results confirm the possibility of varied cancer risks associated with specific variants within the gene. Although the variant-specific risks estimated in our study may be affected by the relatively low statistical power of the tests (reflected in wide 95% CIs) and should be interpreted with caution, this is the first attempt to estimate the risk for distinct *BARD1* PVs. Future studies are required to define the risk more precisely and for a larger set of distinct PVs.

Our results indicate that *BARD1* PVs do not substantially affect OC risk, and a small increase in the OR was not statistically significant (OR = 1.4, *p* = 0.17). The lack of association of *BARD1* PVs with OC risk is consistent with previous observations by Lilyquist et al. (RR = 1.3; *p* = 0.59; *n* = 6294), Ramus et al. (*p* = 0.39; *n* = 3261), and Suszynska et al. (OR = 1.4; *p* = 0.47; *n* = 7063) [1,16,18]. The opposite effect was shown by Norquist et al. (OR = 4.2; *p* = 0.02; *n* = 1915); however, this result should be interpreted with caution, as two out of four carriers were also positive for *BRCA1* PVs [19]. Despite the obtained results, we cannot exclude some very small effect of the PVs on OC risk or the effect of some individual PVs, such as Q564*, which was shown to be associated with a substantial risk of OC.

The findings reported in the study should be interpreted within the context of certain limitations. First, the heterogeneity of the studies (i.e., differences in the inclusion criteria for patients and methodological aspects) and the use of population controls from public databases (not perfectly matched for the geographic area) may affect the results. Second, the association analysis for distinct PVs was performed only for recurrent variants and was of relatively low statistical power. Third, we cannot exclude the presence, in the tested cohorts’ rare cases, of duplicated patients or not reported family members that may increase the recurrence of some variants and have some impact on calculations. Finally, the overall BC and OC risks for *BARD1* were estimated based on loss-of-function variants. The consequences of other variants (including missense variants, silent variants, in-frame deletions/insertions, and multiple non-protein-coding variants) are difficult and/or impossible to define at this time. It is worth mentioning that several of the *BARD1* missense variants have been shown to cosegregate in families with cancer [47,58,78]. Several missense variants in the ANK domain and the BRCT domain were also found to be nonfunctional in the homology-directed repair (HDR) assay; however, most of the tested missense variants did not perturb the HDR function of *BARD1* [149,150,151]. Additionally, an apparent synonymous variant, i.e., R659R, was shown to affect splicing and was suggested to affect cancer risk [143,152]. Due to difficulties in detecting CNV mutations, they are rarely reported in mutation screening studies and therefore were not included in the risk. However, although large CNV mutations are not common in *BARD1* [143], it is unlikely that they do not contribute to BC risk and therefore should be considered in mutation testing. Additionally, it was shown that common variants in *BARD1* that are not the subject of this study may also modify cancer risk, including lung cancer [153] and neuroblastoma [154].

## 5. Conclusions

In conclusion, in this study, we provide a comprehensive summary of *BARD1* PVs, and we provide reliable evidence that *BARD1* is a BC moderate-risk gene but not an OC risk gene. The practical implication of the results is the support that they provide for a substantively justified interpretation of diagnostic results, which could help to establish BC management for *BARD1* PV carriers.

## Supplementary Materials

The following are available online at https://www.mdpi.com/2073-4425/11/7/798/s1, Figure S1. The flow diagram indicating the strategy and criteria used for the selection of articles for the analysis, Table S1. Characteristics of the studies included in the study, Table S2. The list of *BARD1* mutations identified in patients with BC/OC, with their prevalence in BC/OC patients and population controls.
