# Supplementary material for: Summary of BARD1 Mutations and Precise Estimation of Breast and Ovarian Cancer Risks Associated with the Mutations
Source: Genes (Basel). 2020 Jul 15;11(7):798. doi: 10.3390/genes11070798 (PMC7397132; doi:10.3390/genes11070798)
Supplement: Supplementary file 1 [file genes-11-00798-s001.zip › Supplementary Figure and Tables_Suszynska et al_Genes/Supplementary Figure S1_Suszynska et al_Genes.pdf]

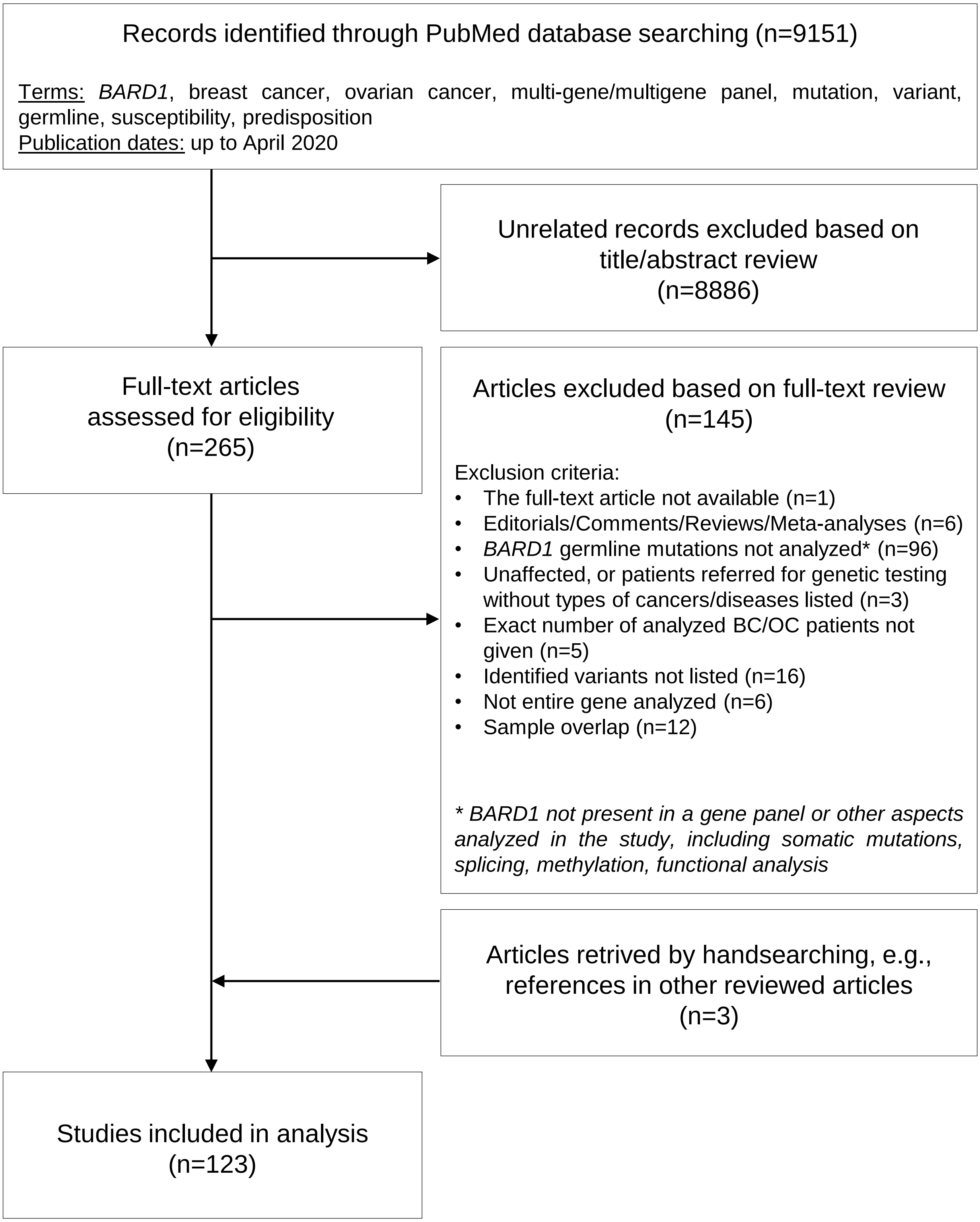

Figure S1. The flow diagram indicating the strategy and criteria used for the selection of articles for the analysis.
